# RETRACTION: Dietary Valine Ameliorated Gut Health and Accelerated the Development of Nonalcoholic Fatty Liver Disease of Laying Hens

**DOI:** 10.1155/omcl/9784052

**Published:** 2025-10-16

**Authors:** Oxidative Medicine and Cellular Longevity

RETRACTION: H. Jian, S. Miao, Y. Liu, et al., “Dietary Valine Ameliorated Gut Health and Accelerated the Development of Nonalcoholic Fatty Liver Disease of Laying Hens,” *Oxidative Medicine and Cellular Longevity* 2021 (2021): 4704771, https://doi.org/10.1155/2021/4704771.

The above article, published online on 25 August 2021 in Wiley Online Library (wileyonlinelibrary.com), has been retracted following the agreement of the journal's Chief Editor, Jeannette Vasquez-Vivar; and John Wiley & Sons Ltd.

The authors initially requested to correct Figure 2e,d, which were duplicated, and Table 2 which contained incorrect primer information.

Further concerns were subsequently identified in Figure 1f where the panels corresponding to 0.74% and 0.79% have overlapping features. As a result of the investigation, the data and the conclusions are considered unreliable, and the article is therefore retracted.

The authors agree with this retraction and the wording of the notice.

